# A Big Data Optimization Approach for Estimating the Time-Dependent Effectiveness Profiles Against Hospitalization for Double- and Single-Dose Schemes: Study Case, COVID-19 in Elderly Mexicans

**DOI:** 10.3390/vaccines13040363

**Published:** 2025-03-28

**Authors:** Óscar A. González-Sánchez, Luis Javier González-Ortiz, María Judith Sánchez-Peña, Humberto Gutiérrez-Pulido

**Affiliations:** 1 Division of Technologies for the Cyber-Human Integration, Universitary Center of Exact Sciences and Engineering (CUCEI), University of Guadalajara, Marcelino García Barragán 1421, Col. Olímpica, Guadalajara CP 44430, Jalisco, Mexico; oscar.gonzalez@academicos.udg.mx; 2 Department of Chemistry, Universitary Center of Exact Sciences and Engineering (CUCEI), University of Guadalajara, Marcelino García Barragán 1421, Col. Olímpica, Guadalajara CP 44430, Jalisco, Mexico; maria.spena@academicos.udg.mx; 3 Department of Mathematics, Universitary Center of Exact Sciences and Engineering (CUCEI), University of Guadalajara, Marcelino García Barragán 1421, Col. Olímpica, Guadalajara CP 44430, Jalisco, Mexico; humberto.gpulido@academicos.udg.mx

**Keywords:** effectiveness profile against hospitalization, COVID-19 vaccines, time-dependent effectiveness, beneficial effect profile against hospitalization, numerical deconvolution, national databases, multidimensional fitting, metaheuristic optimization

## Abstract

**Background:** The COVID-19 pandemic and its handling have made evident the cardinal role of vaccines in controlling the spread of diseases, especially around developed cities. Therefore, precisely characterizing their response has taken a relevant role. Unfortunately, substantial evidence has proven the time dependence of their effectiveness, requiring new approaches that account not only for single value estimations but also for time changes in the effectiveness. **Methodology:** A strategy is proposed to estimate a continuous profile representing the time evolution of the effectiveness against hospitalization. Such a strategy is showcased by characterizing the hospitalization behavior of elderly Mexicans during the COVID-19 pandemic (more than 15 million individuals). **Results**: It is demonstrated that practically total protection against hospitalization can be reached during a noticeable period. However, a substantial depletion in effectiveness occurs after such a plateau. Our methodology provides a continuous profile instead of only a few discrete values, offering insights unattainable by traditional strategies. Furthermore, the obtained profile details allowed for decoupling the effects of each dose independently, enabling the estimation of the expected effectiveness profile for a single-dose scheme. **Conclusions:** The comparison between both schemes (one or two doses) demonstrated that the two-dose scheme is far superior, offering a better investment for public health authorities. Concerning the strategy, the description capabilities of the proposal highly outperform currently available methodologies, allowing for detailed profiles describing the evolution of efficacy to be obtained. This not only opens the opportunity for fair comparison among available vaccines but also creates a tool for researchers studying the immune responses of polydose vaccines.

## 1. Introduction

The COVID-19 pandemic surprised the whole of humanity with a public health challenge never seen before. Like many other sectors of society, the scientific community quickly responded to the quandary. Thus, scientific groups from all over the world systematically attacked the problem from the most diverse fronts. Nowadays, the pandemic is giving us a respite and the scientific community must leverage this period to research and prepare for future challenges, either from this pathology or from other diseases that may spread around the world.

Among diverse efforts, the development of vaccines was one of the most impactful ones in achieving stability. The vaccine technology mRNA-based was the first to provide positive results against the COVID-19 virus and is currently emerging as a promising technology for developing future vaccines [1,2]. Vaccines not only proved to help reduce the number of sick individuals spreading the disease [3,4,5,6,7,8,9,10,11,12,13,14,15] but also were qualitatively estimated to reduce the number of hospitalized patients highly [3,4,5,6,9,10,11,12,13,16,17]. These contributions reduced the pressure and burnout in the healthcare system, allowing the “first line” to handle the situation, significantly reducing the number of deaths.

Collaterally, several policies were implemented to reduce the risk of contagion during the COVID-19 pandemic (e.g., use of face masks, social distancing, air filtration, and increased ventilation in closed spaces, as well as increased use of open spaces). However, vaccines are a valuable strategy that, although they represent significant challenges in their production, the implementation of suitable distribution chains, and the design of vaccine application strategies that discourage contagion, from the vaccine recipients’ point of view, they require little effort compared to isolation policies, offering high levels of protection for substantial periods [3,4,5,6,7,8,9,10,11,12,13,14,15,16,17]. Furthermore, critical sectors of the population (e.g., healthcare, energy, food logistics, communications) cannot easily suspend operations, as they are fundamental for societies. Thus, strategies like vaccination to protect them are required.

Nevertheless, before any massive vaccination process starts, vaccines must validate their safety and effectiveness through rigorous tests [18,19]. Traditionally, such tests have been performed through clinical trials. Such trials [3,4,5,6,7,8,9] aim to identify and eliminate any adverse effects that may occur in the population (thus ensuring safety) while additionally estimating their effectiveness. Even though this kind of study ensures safety and suggests the effectiveness of the vaccine, it has limitations in the precision of the estimated effectiveness (due to the small number of events considered, typically around a couple of hundred confirmed cases), exhibiting comparatively large confidence intervals [3,4,5,6,7,8,9].

In addition, assuming that effectiveness is maintained constant for considerable periods, the confirmed cases during the whole study are globally counted to calculate a single effectiveness value with a confidence interval assumed to be valid for the entire study period, highly affecting the precision [3,4,5,6,7,8,9]. Moreover, the last methodology cannot be applied to vaccines with time-dependent effectiveness as the biases increase, and, in some cases, strong decrements in vaccine effectiveness could be overlooked [20].

Considering the above, several scientific groups have expanded the results from the original phase three clinical trials by evaluating the performance of the vaccines once they have been broadly applied to societies, typically grouping the confirmed cases in monthly evaluations [10,11,12,13,14,15,16,17]. This procedure highly improves the precision of the estimations of the effectiveness of the vaccines and allows for decay trends to be detected. However, such periodic evaluations are far from producing a smooth profile that clearly indicates the behavior of the vaccines in the recipients. In addition, this methodology implicitly considers that the vaccine effectiveness remains constant during the study period (usually ≥1 month), which is conceptually incorrect and inevitably induces biases.

Thus, in a previous paper [21], we developed a mathematical optimization strategy to obtain the continuous effectiveness profile against symptomatic disease, which characterized the set of vaccines applied to elderly Mexicans. This procedure used information about more than one million confirmed cases (more than 1000 times more confirmed cases than the typical phase 3 clinical trials [3,4,5,6,7,8,9]). Such an enormous amount of information allowed the obtainment of a continuous global profile of effectiveness against symptomatic disease, portraying the daily effectiveness values that the vaccine exhibited as time elapsed since the last dose application increased.

Furthermore, such a procedure also allowed for decoupling the effects induced by each dose, allowing the estimation of a better period between shots to enlarge the protection obtained [21]. Later, employing a target trial emulation approach, another research group pursued a similar objective, obtaining qualitatively equivalent trends [22]. Additionally, the proposal presented in [21] opened the door for the implementation of close follow-ups of millions of individuals to generate detailed profiles with valuable insights for further research.

The continuous profiles offer several advantages concerning their discrete counterparts, allowing, for example, the disengagement of the effects of the first and second doses, the optimization of the time between shoots to increase the protection period, making early detections of decay in protection (suggesting the necessity of booster applications), or even the early screening of strains that may affect the effectiveness of the currently distributed vaccine.

Considering the above, this manuscript represents the next step in characterizing continuous effectiveness profiles, adapting our previous proposals [20,21] to be able to handle not only profiles against symptomatic disease but to estimate effectiveness against hospitalization (a more complex system with fewer cases). Furthermore, the global effectiveness profile against hospitalization estimated for the two-dose scheme is decoupled into the effects of each dose and joined with the independent effects against symptomatic disease to allow for the estimation of the hospitalization protection profiles of the single-dose and two-dose schemes, enabling a quantitative comparison between both of them.

Thus, the remainder of this manuscript is organized as follows: Section 2.1 describes the obtainment procedure of the real-world profiles required to estimate the effectiveness profile against hospitalization (vaccination profile—Section 2.1.1 and beneficial effect profile—Section 2.1.2), detailing the adaptations that must be considered to apply the previously developed strategy [21] to the hospitalization process. In Section 3, the obtained results are presented. Thus, the estimation of the global effectiveness profile for the two-dose schemes, as applied to the 60+ group, is made in Section 3.1. Complementarily, the effectiveness profile for the hypothetical single-dose scheme is estimated in Section 3.2, including a comparison between the effectiveness behavior estimated for the single-dose and two-dose schemes. Then, a discussion of the results and their limitations is presented in Section 4. Finally, Section 5 presents the manuscript’s conclusions, remarking on the noticeable advantages of the proposed methodology.

## 2. Methodology

The procedure for obtaining the effectiveness profile against hospitalization for the global scheme (two doses) starts with obtaining, from information included in real-world databases, the vaccination profile and the beneficial effect profile. A numerical deconvolution process must then be applied between such profiles to obtain the effectiveness profile that better correlates such profiles. Finally, an endpoint must be selected for the effectiveness profile based on the standard deviation between numerical solutions. Such an endpoint allows for the partition between the robust estimation period for the available data (desired profile) and the period showing blurry estimations that must be neglected (artifacts generated at the end of signals by numerical deconvolution).

For the case of the single-dose profile, an additional decoupling procedure is also required to find two independent profiles that, following an interaction model, better match the global effectiveness profile against hospitalization. As many profiles could satisfy such a general constraint, a vaccine response model must be employed. This work considered a general response model developed for COVID-19 vaccines in a previous article [21], achieving a great fit between the modeled and real-world data profiles.

### 2.1. Required Real-World Profiles

This proposal employs a mathematical operation from the signal processing field known as convolution to estimate the continuous evolution of the effectiveness against hospitalization. Convolution is a fundamental operation that has been recently adapted [20] to characterize how the effectiveness of vaccines (E[k]; distorting media) interacts with the distribution and timing of vaccination efforts (f[t]; original stimulus signal) to produce a reduction in the incidence of confirmed cases (B[t]; observable effects in the system) as follows:

(1) $$B\left[t\right]=\left(f*E\right)\left[t\right]=\sum_{n}f\left[t-k\right]E\left[k\right]$$

Additional details can be read in our previous papers [20,21].

Thus, as two of the three profiles involved in Equation (1) could be obtained from national databases [23,24,25], this allows for solving the third one, in this case, the time-dependent effectiveness profile.

#### 2.1.1. The Vaccination Profile

Regarding the vaccination profile ($f\left[t\right]$), it can be typically obtained from public health institutions. For the showcased example of this study, the profile obtainable from the publications of the Government of Mexico Health Secretary [23,24] was employed, as described in references [20,21]. This profile comprises a set of daily percentages representing the number of individuals with ≥60 years vaccinated daily with any of the approved vaccines in Mexico [23,24] (for reproducibility easiness, the numeric values of such a profile are presented in Supplementary Material S1); daily percentages were calculated considering that 60+ group included 15,121,683 elderly Mexicans. Thus, more than 10 million Mexicans in this group were vaccinated and most of them were vaccinated with two-dose schemes of the BNT162b2 (Comirnaty^®^; Pfizer; New York, NY, USA) vaccine [3] or two-dose schemes of the AZD1222 (Vaxzevria^®^, AstraZeneca; Cambridge, UK) vaccine [4].

#### 2.1.2. The Beneficial Effect Profile

Concerning the beneficial effect profile ($B\left[t\right]$), additional considerations must be taken into account to adapt the original procedure to hospitalization instead of symptomatic disease [21]. Originally, the proposed strategy [21] was used to obtain the beneficial effect from the daily incidence ratio between the 60+ group ($I_{study\;group}\left[t\right]$) and the 30–39 group ($I_{reference\;group}\left[t\right]$), following the expression of Equation (2).

(2) $$B\left[t\right]=1-\frac{I_{study\;group}\left[t\right]}{I_{reference\;group}\left[t\right]}$$

Nevertheless, the validity of Equation (2) requires an equal probability of infection between the studied (60+) and reference (30–39; total population of this group: ~18.4 million) groups during the unvaccinated period; for symptomatic disease, this requirement was fulfilled by Mexicans older than 30 years [20,21].

**Figure 1:**
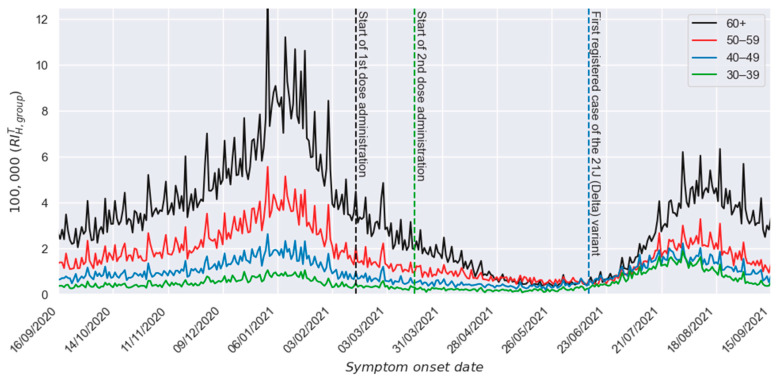
Incidence of daily hospitalized individuals per 100,000 members of each group.

However, this requirement cannot be identically applied between the hospitalization groups, as a hospitalized individual with COVID-19 must be symptomatic first, adding a composed probability between procedures. In addition, individuals grouped by age experience different affectations once infected, as the disease effects are highly influenced by age. This phenomenon can be evidenced by analyzing in Figure 1 the hospitalization relative incidences of the different groups. Figure 1 also includes the following dates: (a) the start of the first dose administration, (b) the start of the second dose administration, and (c) the date of the first registered case of the 21J (Delta) variant in Mexico [26]. In addition, it is important to mention that the period from April 1 to 15 September 2021, was considered in estimating the effectiveness profile. In such a period, 181,895 individuals of the 60+ group and 35,789 individuals of the 30–39 group were hospitalized.

In Figure 1, it can be appreciated that the respective profiles for relative incidences of the study group (60+) and the reference group (30–39) show qualitatively similar shapes during the unvaccinated period (left side of vertical black line). However, they do not overlap, as occurred with the relative incidences of confirmed cases [20]. As mentioned, the hospitalization behavior is determined by the increased sensibility to the virus shown by infected individuals as their ages increase.

**Figure 2:**
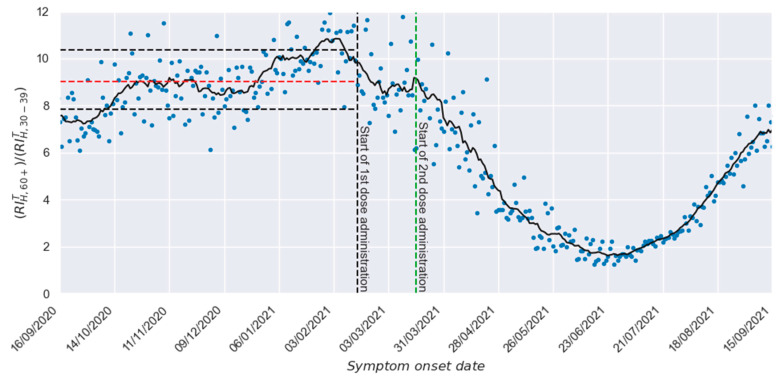
Quotient between the relative incidence of hospitalized cases between reference and study groups.

Nevertheless, a more in-depth analysis can be carried out using Figure 2. There, the quotient between the relative incidences of study (60+) and reference (30–39) groups is presented, making evident that, during the unvaccinated period, such quotient is around 9. The dotted red line in Figure 2 denotes the average value of the real-world relative incidence values (blue points) between 16 September 2020 and 15 February 2021 (153 daily values), and, as reference marks, the horizontal black lines show a deviation of 15% concerning such average value. A variability level in the order of 15%, as shown by the continuous black line during the unvaccinated period (smoothed line obtained using a centered moving average of 15th order [27]), is expected in a social phenomenon like the one considered here.

Nonetheless, after starting the second dose administration (right side of the vertical green line), it is also possible to observe a strong and consistent decrement in the relative incidence of hospitalization in the study group, which shows the strong protection response generated by the vaccination process. Unfortunately, the waning on the effectiveness of administrated vaccines determined that such a favorable trend ended in July of 2021. Although such a waning could be originated, at least partially, by the waning of the humoral response generated by the vaccine in the involved recipients [28,29,30,31], since the proposed methodology neither considers information about the humoral responses of involved individuals nor provides information about such parameter, the possible correlation between the humoral response and the vaccine effectiveness is beyond the scope of the present work. An alternative option to explain such a waning is the emergence of comparatively more resistant variants. Unfortunately, the absence of enough precise information about the distribution of variants in Mexico prevents us from establishing statements on this topic.

**Figure 3:**
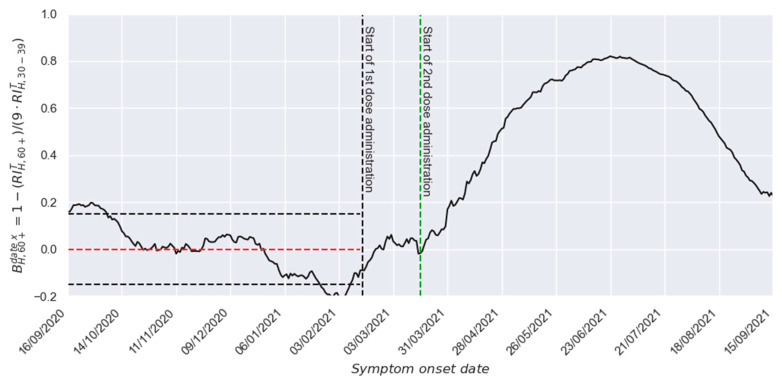
Beneficial effect profile against hospitalization after applying the required adaptations for hospitalization characterization.

In those circumstances, to adapt the original deconvolution procedure to the hospitalization phenomena, it is necessary to consider that the infections in the 60+ group produced nine times more hospitalizations than those in the 30–39 group. Thus, Figure 3 presents the B[t] profile generated considering such a normalization process (for reproducibility easiness, the numeric values of such a profile are also presented in Supplementary Material S1). There, it is possible to observe a B[t] value around zero before the vaccination process starts (since vaccination was not started, no beneficial effect is produced) and a strong response afterward. Furthermore, it is also possible to observe that such beneficial effect increases for almost 4 months due to the gain in effectiveness in each recipient and the increment in the number of vaccinated individuals (the vaccination process in the 60+ group lasted 78 days). Finally, it is also possible to observe that the beneficial response starts a noticeable waning after such an increase, strongly demonstrating a substantial loss of protection in the population after a couple of months.

## 3. Results

The results mainly focus on obtaining the effectiveness profile against the hospitalization that characterizes the study group. However, due to the high capabilities of the used algorithm, it was possible to disengage the effects of the first and second dose, allowing us to propose the global effectiveness profile for the two-dose scheme, as it was applied to the 60+ group, but also the expectable effectiveness profile for a hypothetical administration of a scheme with only one dose, as shown below.

### 3.1. Two-Dose Scheme

Once the required profiles have been properly adapted (Section 2.1), it is possible to execute a deconvolution to find the effectiveness profile that better correlates the inputs of the system (vaccinations) with the outputs (beneficial effect observed). However, as described in the previous paper [21], deconvolution is highly sensitive to noise (e.g., measurement errors or random fluctuations). Therefore, several optimization procedures are required to be generated and averaged to find an appropriate solution. Concerning vaccine characterization, as in our previous work [21], the Particle Swarm Optimization algorithm (PSO [32,33,34]) was tailed to this specific case to obtain a PSO-based optimization approach (PSOSS from now on; Particle Swarm Optimization for Smooth Solutions). The PSOSS algorithm allows the fitting of the effectiveness profile not to be arbitrarily regulated, as no initialization or functionality is predefined, enabling the metaheuristic algorithm to reconstruct, without any restriction, the transfer function required to satisfy the equality expressed in Equation (1). Moreover, as metaheuristic algorithms are not deterministic, the strategy used considers, as the proposed result, the mean of 1000 estimations, allowing the multiple estimations to converge into a general consensus profile that collects the findings of each optimization process.

Such a metaheuristic optimization approach will find an optimized solution through iterative improvement, looking to minimize the deviation between the real-world $B\left[t\right]$ profile and a modeled one ($B_{model}\left[t\right]$) generated by convoluting the real-world $f\left[t\right]$ and a proposed $E\left[k\right]$ profile as described by Equation (1). Thus, by iteratively improving the profile through a set of 1000 epochs, it is possible to achieve a fit that deviates the least from the real-world data. Concerning such a 1000-epoch procedure, a video is provided in Supplementary Material S2 showing the iterative fitting of the 1000 numerical proposals as iterations increase, presenting simultaneously the effectiveness candidate profile and the corresponding $B_{model}\left[t\right]$ implied.

After such a procedure has been implemented, an endpoint to the deconvoluted signal must be set, employing an elbow/knee condition based on the standard deviation across solutions [35]. For the example showcased in this manuscript, an endpoint at 135 days after the second dose administration was selected, considering the consistency of the solutions before such threshold. On this topic, it is convenient to mention that the proposed methodology requires that the reference group (in this case, the 30–39 group) remain entirely unvaccinated. Therefore, only information about hospitalized individuals showing symptoms onset before 15 September 2021 was considered. Thus, during the first fortnight of September, the number of individuals who had more than 135 days after complete vaccination was comparatively low. Due to this, estimations become blurry for periods longer than 135 days since second dose administration.

**Figure 4:**
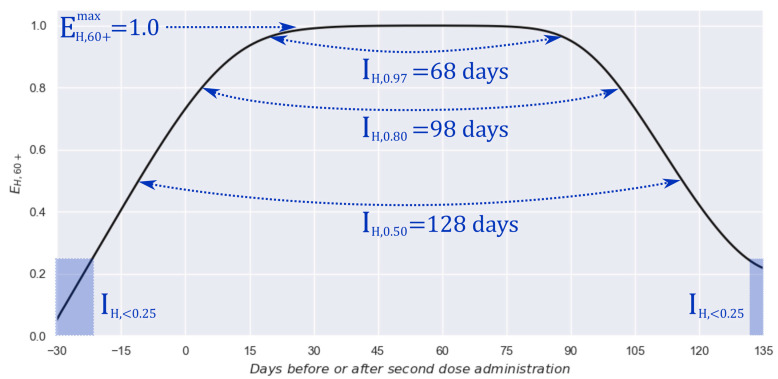
Estimated effectiveness profile against hospitalization for the 60+ group and graphical definitions for some relevant parameters: (a) maximum effectiveness: $E_{H,60+}^{max}$, (b) interval with effectiveness practically total: $I_{H,0.97}$, (c) interval with excellent effectiveness: $I_{H,0.80}$, (d) interval with acceptable effectiveness: $I_{H,0.50}$, and (e) intervals with poor effectiveness: $I_{H,<0.25}$.

Thus, Figure 4 presents the resulting profile from this procedure. Additionally, several interest parameters are added through markers to ease the profile’s interpretation and highlight valuable information.

By analyzing Figure 4, it is possible to observe that the maximum effectiveness against hospitalization is, astonishingly, 100%, and it is preserved at acknowledgeable high values (above 97%) for approximately 2 months (68 days). Moreover, it is kept at high values (above 80%) for approximately 3 months (98 days) and at “in-service” values (above 50%) for about 4 months (128 days). Finally, it is possible to observe a quick response, achieving an effectiveness of 25% 3 weeks before the second dose administration. In addition, a possible start of the end of protection (effectiveness < 25%) can be located after day 130.

**Figure 5:**
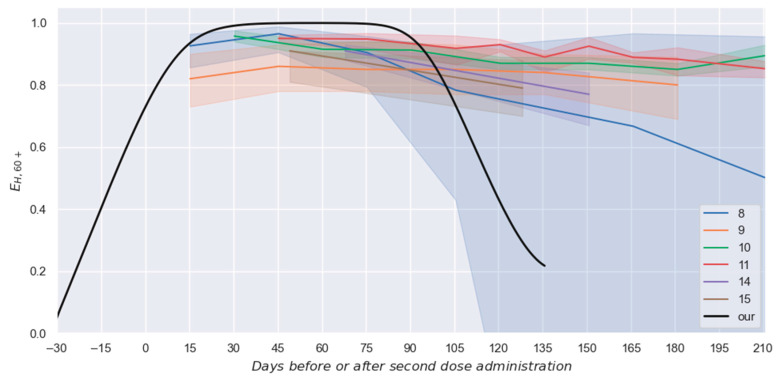
Comparison between the proposed profile and discrete intervals for the effectiveness against hospitalization reported in six different studies [10,11,12,13,16,17].

Nevertheless, it is important to contextualize this profile with other estimations achieved by research groups worldwide. Therefore, Figure 5 presents the estimated profile in conjunction with several discrete estimations currently available [10,11,12,13,16,17], considering their respective confidence intervals for all cases (including the imperceptible interval achieved by our proposal).

### 3.2. Single-Dose Schemes

In the previous section, the effectiveness profile against hospitalization for the two-dose scheme (set of $E_{H,60+}\left[k\right]$ values) was obtained. The practical usefulness of such a set of values is undeniable, as it characterized the effectiveness of the two-dose schemes as they were applied to the population. However, the estimation of the profile for the effectiveness in preventing hospitalizations among symptomatic individuals ($E_{H|S,60+}\left[k\right]$) is also useful from a medical point of view and/or for research purposes, as it allows for the details of the effect of each dose to be clearly visible. To attain such an aim, it is possible to employ the Bayes theorem [36] to correlate the set of $E_{H|S,60+}\left[k\right]$ values, with the effectiveness profile against the hospitalization (set of $E_{H,60+}\left[k\right]$ values) and the effectiveness profile against the symptomatic disease ($E_{S,60+}\left[k\right]$), as indicated in Equation (3).

(3) $$E_{H|S,\;60+}\left[k\right]=1-\frac{1-E_{H,\;60+}\left[k\right]}{1-E_{S,60+}\left[k\right]}$$

A formal demonstration of Equation (3) is presented in Supplementary Material S3. Note that such a relationship can be equivalently applied to two-dose schemes or single-dose schemes.

For the two-dose systems, since the set of $E_{H,60+}\left[k\right]$ values were estimated in the previous section, and the set of $E_{S,60+}\left[k\right]$ values were reported in our previous paper [21], Equation (3) allows us to obtain the set of $E_{H|S,60+}\left[k\right]$ values.

**Figure 6:**
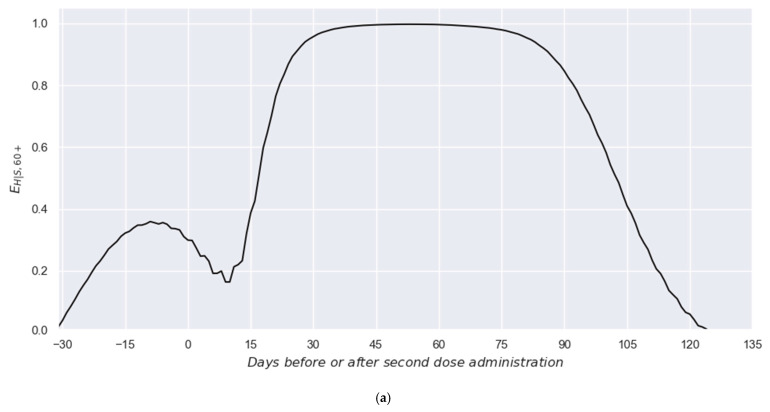

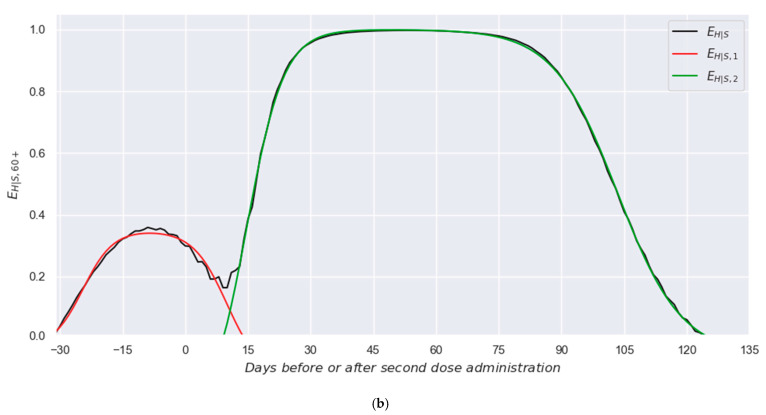
Estimated effectiveness profiles against hospitalization among symptomatic individuals. (**a**) Effectiveness profile in preventing hospitalizations among symptomatic individuals. (**b**) Independent effectiveness profiles for the first (in red) and second (in green) doses.

Thus, Figure 6a presents the obtained profile for the effectiveness in preventing hospitalizations among symptomatic individuals ($E_{H|S,60+}\left[k\right]$). There, it is possible to observe a clear bimodality generated by the effects of the first and second doses. Thus, although the set of $E_{H|S,60+}\left[k\right]$ values do not represent the traditional effectiveness against hospitalization (as the $E_{H,60+}\left[k\right]$ does), such a profile allows for the independent effects of each dose to be evidenced, making the mathematical separation of such effects easier, which is not possible with the $E_{H,60+}\left[k\right]$ profile alone.

Once the set of $E_{H|S,60+}\left[k\right]$ values is available, it is possible to employ the interaction model (Equation (4)) and a response model (Equation (5)) used in our previous paper [21] to separate the effects of each dose. Such interaction and response models are considered here due to their generality and proven capacity to properly decouple the independent effects of each dose in the two-dose schemes administrated to the 60+ group [21].

(4) $$E_{H|S,60+}\left[k\right]=E_{H|S,60+,1}\left[k\right]+Q\left(E_{H|S,60+,2}\left[k+∆k\right]\right)$$

(5) $$E_{H|S,60+,1\;or\;2}\left[m\right]=\left(\left(A_{L}\right)\left(\frac{e^{\left(m+D_{L}\right)\left(C_{L}\right)}-e^{-\left(m+D_{L}\right)\left(C_{L}\right)}}{e^{\left(m+D_{L}\right)\left(C_{L}\right)}+e^{-\left(m+D_{L}\right)\left(C_{L}\right)}}\right)+B_{L}\right)\left(\left(A_{R}\right)\left(\frac{e^{\left(m+D_{R}\right)\left(C_{R}\right)}-e^{-\left(m+D_{R}\right)\left(C_{R}\right)}}{e^{\left(m+D_{R}\right)\left(C_{R}\right)}+e^{-\left(m+D_{R}\right)\left(C_{R}\right)}}\right)+B_{R}\right)$$

In Equation (4), the $E_{H|S,60+}\left[k\right]$ (set of values characterizing the two-dose profile) and $E_{H|S,60+,1}\left[k\right]$ (set of values representing the effect of the first dose) parameters start their respective effects at the same time. Concerning the $E_{H|S,60+,2}\left[k+∆k\right]$ parameter, it is an effectiveness profile representing only the beneficial effect induced in recipients by the second dose. Since such an effect starts later, specifically ∆k days later, in the adopted notation, the functionality with time must be expressed as k+∆k. Due to its demonstrated flexibility (see the video in Supplementary Material S4a and S4b of reference [21]), Equation (5) was selected as the response model. In fact, our previous papers [20,21] also used such an equation to model the effectiveness behavior of vaccines administrated to the population group here considered. Additional details can be found in our previous publication [21].

Thus, since the set of $E_{H|S,60+}\left[k\right]$ values, Equations (4) and (5), and a general optimization algorithm (for this case, the GRG Nonlinear optimizer from Microsoft Excel was used [37,38,39]) are available, the two independent profiles can be separated; that is, the profile representing the effect of the first dose on the effectiveness against the hospitalization of symptomatic individuals ($E_{H|S,60+,1}\left[k\right]$; red line in Figure 6b) and the one representing the effect of the respective second dose ($E_{H|S,60+,2}\left[k+∆k\right]$; green line in Figure 6b). There, it is possible to observe the great fit achieved by this flexible response model, which was demonstrated to be adaptable to the shapes of the individual effects.

Now, we will focus on the single-dose systems, analyzing the expected behavior when only the effect of the first dose is considered. Thus, it will be reconsidered Equation (3) applying it to the single-dose system and solving for the $E_{H,60+,1}\left[k\right]$ parameter as follows:

(6) $$E_{H,60+,1}\left[k\right]=1-\left(1-E_{H|S,60+,1}\left[k\right]\right)(1-E_{S,60+,1}\left[k\right])$$

**Figure 7:**
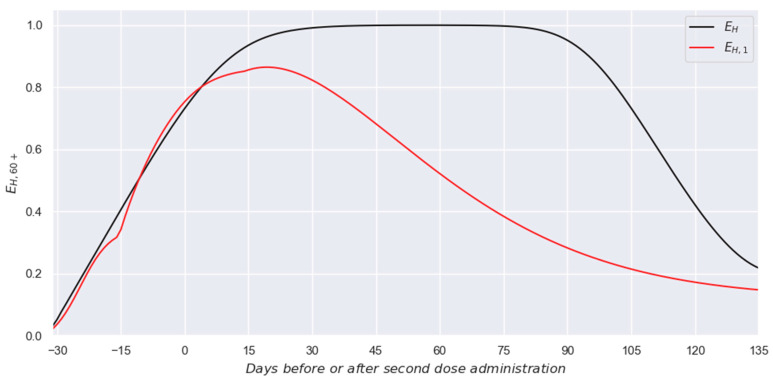
Comparison between the single-dose and two-dose schemes against hospitalization.

In this case, since the set of $E_{H|S,60+,1}\left[k\right]$ values have been previously estimated (red line in Figure 6b) and the set of $E_{S,60+,1}\left[k\right]$ values were reported in our previous paper [21], Equation (6) allows us to estimate the desired profile for the effectiveness against hospitalization expected when only the first dose is administrated ($E_{H,60+,1}\left[k\right]$ profile). The resulting $E_{H,60+,1}\left[k\right]\;$ profile is presented in Figure 7. Additionally, for comparison purposes, the equivalent profile for the two-dose scheme is also presented in such a figure ($E_{H,60+}\left[k\right]$ profile).

## 4. Discussion

The global challenge generated by the COVID-19 pandemic promoted several changes and responses in the different population sectors. However, the scientific community was one of the most active sectors, offering solutions to face the quandary from diverse perspectives. Furthermore, vaccine developments allowed for the quick deployment of vaccines, highly reducing the affectations and the number of deaths.

Nevertheless, despite no effort being spared concerning effectiveness estimation, available strategies only allowed for discrete effectiveness values to be obtained at that time. However, several independent research groups later proved the time dependency of the effectiveness of several vaccines, promoting the development of methods to follow the evolution of the effectiveness as time since the last dose administration increases.

Thus, this work proposes a strategy to estimate the continuous effectiveness profile against hospitalization. Such a strategy improved their counterparts by offering a smooth continuous profile instead of several discrete values while also reducing the confidence intervals of the estimation by considering several millions of individuals to make the inferences by employing national databases.

Such a reduction in the confidence intervals can be observed in Figure 5, where our estimation has a confidence interval so slim that it is covered by the black line presented in such a figure.

Nevertheless, although the proposed strategy could be employed to improve the estimation of vaccine effectiveness against hospitalization, some elements of the showcased example must be considered before performing effectiveness comparisons among the information presented in Figure 5. Thus, it is relevant to mention that, excepting a work [17] that studied a group that was vaccinated with BNT162b2 or mRNA-1273 vaccines (brown area), the other studies considered only individuals vaccinated with BNT162b2. Additionally, it must be mentioned that in one study [10] (blue area), the reported values represent the global effectiveness against hospitalizations and death, while the rest [11,12,13,16,17] present only the effectiveness against hospitalization. In addition, in reference [16] (purple area), the reported effectiveness values are for adults, while in reference [10] (blue area), the studied group included only individuals older than 60. In contrast, in the other four studies [11,12,13,17], the studied population was people aged 65 or more. Thus, although considerable similarities exist between the experimental systems previously considered and the here considered (60+ group mostly vaccinated with BNT162b2 or AZD1222 vaccines [20]), a quantitative comparison cannot be performed.

Considering those mentioned above, it could be affirmed that there is a qualitative agreement between the previous results and the proposed profile here. The main differences among them occur at times higher than 4 months after the second-dose administration. During such a period, the previously reported effectiveness values are usually higher than the estimated by this work [11,12,13,16,17]. However, such behavior could be explained by considering the effect of the inclusion in the studied group of individuals vaccinated with vaccines different than the BNT162b2. Note that around two-thirds of the vaccinated individuals in the 60+ group received the BNT162b2 vaccine (~7 million individuals), while most of the rest of the vaccinated individuals in this group received AZD1222 vaccines (few more than 2 million persons). In this topic, it is important to note that the reported interval for the effectiveness against the symptomatic disease of the AZD1222 vaccines (CI 95%: 54.2–94.1% [4]) is considerably lower than the one characterizing the BNT162b2 vaccines (CI 95%: 66.7–99.9% [3]). It would be preferable to perform the previous comparison considering the effectiveness against the hospitalization process instead of the one for the symptomatic disease. Unfortunately, the short duration of the clinical trials, combined with the comparatively low number of individuals studied in the respective phase 3 clinical trials, prevented the obtainment of acceptably precise values for such a parameter [3,4].

Nevertheless, the proposed strategy provides insights unachievable by the alternative strategies, for example: (a) results having an almost unnoticeable confidence interval (due to the more than 33 million considered individuals and 217,684 hospitalized individuals taken into account), (b) the proposed profile provides information since the day of the first dose application, showing the early immune responses of the recipients, (c) the proposed methodology avoid some biases characterizing the other proposals (e.g., those produced by the incorrect grouping of events over extended timeframes), (d) our profile follows a smooth and natural contour in contrast to the shaky and inconsistent values estimated by the monthly grouping proposals, and (e) the high realism level of the estimated profile allows the decoupling of the individual effects of each dose (Figure 6), which is only possible with smooth contours showing bimodalities or characteristics that enable the disengaging of the individual effects of each dose (Section 3.2).

In addition, concerning the comparison between the double-dose and single-dose profiles, it is essential to note that effectiveness must be analyzed as reductions in risk. Therefore, a change in effectiveness from 50-ish% values to 95-ish% values does not imply a double in effectiveness, but a tenfold reduction in risk. Thus, the presented profiles in Figure 7 imply that the two-dose scheme is far superior to the single-dose scheme, achieving higher effectiveness values with astonishing implied immunities. Moreover, it generates a lasting immune response that firmly holds the effectivity at high values (with a long plateau instead of just a cusp) while practically doubling the service period (period with effectiveness values higher than 50%).

Considering the above, one can easily conclude that, from the social and vaccinated individuals’ point of view, the two dose schemes are highly preferable to the single ones due to the astonishing protection level. Additionally, it must also considered that, from an economic and production point of view, the two-dose schemes (at least for these vaccines) generate a better return on investment than single-dose schemes, thus making them preferable for minimizing the number of hospitalizations whenever scarcity allows it.

Nevertheless, it is important to note that the above-mentioned descriptions are valid for the system considered. The relative importance of the effect of one dose compared to the complete two-dose vaccination scheme depends on the type of vaccine administered. In fact, for vaccines designed to be administered as a two-dose scheme, administering only the first dose could be anticipated to provide comparatively poor protection. However, in schemes designed to administer only one dose, the second dose could act like a booster. For this new case, comparing the first and both doses could result in a considerably different behavior than the one described here. Thus, the here presented results cannot be quantitatively extrapolated to other vaccines; each case must be studied individually.

Additionally, it is expected that the changes in the daily effectiveness values can be correlated to the set of modifications in the humoral immune response occurring in each recipient (e.g., changes in the binding and functional antibodies) [28,29,30,31]. However, the proposed methodology estimates the effectiveness profile considering the social effect produced, that is, the relative decrease in the number of hospitalizations in the vaccinated group compared to the number of hospitalizations in the reference group under equivalent conditions. Thus, it does not require knowledge of the temporal evolution of the humoral changes produced in the recipients, nor can it provide any information about these changes. Nevertheless, the results presented here can be used to estimate, in future work, the correlation between the effectiveness profile and the time evolution of the humoral response in recipients.

Finally, it is important to mention that the estimations obtained in this work represent the global behavior of the pool of vaccines administrated to the 60+ group. Estimating the individual effects of each vaccine type administrated to the 60+ group would be desirable. However, since unavailable information is required to disengage the individual effects of each vaccine type, such valuable information cannot be reported here.

## 5. Conclusions

This work proposed a procedure to estimate a continuous profile representing the evolution of the effectiveness against hospitalization in a given population group. The proposed methodology was showcased with data from the elderly Mexicans (60+ group). The results demonstrate that, for the example case, practically total protection against hospitalization was achieved during a noticeable period, but such a protection level was far from permanent. Additionally, the superiority of our results was demonstrated, offering a precise continuous profile with a remarkably low confidence interval that removed several biases generated by the characterization of only a few discrete effectiveness values, as traditionally made. In addition, the high capabilities of the proposal allowed us to portray fine details in the effectiveness profile, allowing for the decoupling of the effects of the first and second doses. Such decoupling enabled the estimation of the expected effectiveness profile for a single-dose scheme. The comparison between the effectiveness behavior of the single-dose and two-dose schemes demonstrated that the two-dose scheme is far superior to the single-dose scheme, offering valuable information for public health decision-makers who must ponder the best solutions concerning their available resources. Thus, this proposal could be helpful for several research lines that range from improving vaccination schedules, aiming to maximize the period for which a desired effectiveness is held, to studying in detail the effectiveness profiles of vaccines against death.

## Data Availability

The basic data used for performing this study are available in the links referenced in this article’s main text or the files included in its Supplementary Materials.

## Supplementary Materials

The following supporting information can be downloaded at: https://www.mdpi.com/article/10.3390/vaccines13040363/s1, Supplementary Material S1: Excel spreadsheet showing the numerical values of the required vaccination profile and those describing the used $B_{H,60+}$ profile; Supplementary Material S2: Video simultaneously showing the iterative modification of the $E_{H,60+}$ profile as iterations increase, and the respective implied estimation of the $B_{H,60+}$ profile. Supplementary Material S3: Demonstration of Equation (3).
