# Supplementary material for: A Big Data Optimization Approach for Estimating the Time-Dependent Effectiveness Profiles Against Hospitalization for Double- and Single-Dose Schemes: Study Case, COVID-19 in Elderly Mexicans
Source: Vaccines (Basel). 2025 Mar 28;13(4):363. doi: 10.3390/vaccines13040363 (PMC12031172; doi:10.3390/vaccines13040363)
Supplement: Supplementary file 1 [file vaccines-13-00363-s001.zip › Supplementary Material S3.pdf]

The relationship between the effectiveness against the hospitalization process and the effectiveness against hospitalization among symptomatic individuals

The classical definition used to calculate the effectiveness against the hospitalization process (E_H) for a vaccine showing a constant value of effectiveness for a very long time (no time-dependent effectiveness), is shown below:

$$E_H = 1 - \frac{RI_H^V}{RI_H^U} = 1 - \frac{\frac{H^V}{P^V}}{\frac{H^U}{P^U}} \quad \text{(SM3-1)}$$

In Equation SM3-1, RI_H^V and RI_H^U are the relative incidences of hospitalized individuals on the respective fractions vaccinated (v) and unvaccinated (u) of the study group. Each of such relative incidences represents the quotient between the number of hospitalized individuals in the respective fraction (vaccinated -H^V- or unvaccinated -H^U-) in a given period (usually around a hundred days) and the respective number of members included in each fraction (P^V or P^U).

However, Equation SM3-1 can be adapted for a vaccine showing time-dependent effectiveness, considering that such types of vaccines exhibit a different effectiveness value each day. To make the mathematical process more manageable, let us consider that all individuals were vaccinated on the same day in this example. Thus, to obtain the effectiveness value ($E_H[k]$) of a vaccine k days after the second dose administration (from now on, time-from-vaccination), it is required to consider that the period to count the number of hospitalized individuals is a day. Thus, the H^V parameter becomes the $H^V|^{date\ x}$ parameter, and the H^U one becomes the $H^U|^{date\ x}$ one. Similarly, the P^V parameter becomes the $P^V|^{date\ x}$ parameter, and the P^U one becomes the $P^U|^{date\ x}$ one. Therefore, the notation for the respective relative incidences must be modified as indicated in Equation SM3-2.

$$E_H[k] = 1 - \frac{RI_H^V|^{date\ x}}{RI_H^U|^{date\ x}} = 1 - \frac{\frac{H^V|^{date\ x}}{P^V|^{date\ x}}}{\frac{H^U|^{date\ x}}{P^U|^{date\ x}}} \quad \text{(SM3-2)}$$

Thus, the set of $E_H[k]$ values (one for each k value) constitute the time-dependent effectiveness profile against the hospitalization process (E_H profile). The SM3-2 expression provides a set of effectiveness values ($E_H[k]$ values) characterizing the behavior of the vaccines administrated to those individuals having at the $date\ x$ a time-from-vaccination of k days. It is important to remark that when

a given type of vaccine exhibits no time-dependent effectiveness, the $E_H[k]$ value, as well as the $RI_H^V|^{date\ x}/RI_H^U|^{date\ x}$ quotient remains constant over time and the expression SM3-2 can be simplified, eliminating the k and $date\ x$ notations. Thus, the classical definition of the vaccine effectiveness against the hospitalization process (Equation SM3-1) is a particular case of Equation SM3-2, which is only valid for vaccines exhibiting no time-dependent effectiveness.

Nevertheless, when explicitly applied to the 60+ group, Equation SM3-2 can be reordered and algebraically modified as follows:

$$1 - E_{H,60+}[k] = \frac{RI_{H,60+}^V|^{date\ x}}{RI_{H,60+}^U|^{date\ x}} = \frac{\frac{H_{60+}^V|^{date\ x}}{P_{60+}^V|^{date\ x}}}{\frac{H_{60+}^U|^{date\ x}}{P_{60+}^U|^{date\ x}}} = \left(\frac{\frac{H_{60+}^V|^{date\ x}}{CC_{60+}^V|^{date\ x}}}{\frac{H_{60+}^U|^{date\ x}}{CC_{60+}^U|^{date\ x}}}\right)\left(\frac{\frac{CC_{60+}^V|^{date\ x}}{P_{60+}^V|^{date\ x}}}{\frac{CC_{60+}^U|^{date\ x}}{P_{60+}^U|^{date\ x}}}\right) \qquad \text{(SM3-3)}$$

In the Equation SM3-3, the $CC_{60+}^V|^{date\ x}$ and $CC_{60+}^U|^{date\ x}$ parameters are the number of confirmed cases having a symptoms' onset date on the $date\ x$, counted in the fraction vaccinated (v) or unvaccinated (u) of the 60+ group, respectively.

Additionally, following a procedure similar to the previous one, the classical expression for the effectiveness against symptomatic disease ($E_S = 1 - \frac{RI^V}{RI^U} = 1 - \frac{\frac{CC^V}{P^V}}{\frac{CC^U}{P^U}}$)) can be adapted to be applied to vaccines exhibiting time-dependent effectiveness by including, as in the previous procedure, the k and $date\ x$ notations. Thus, for the 60+ group, the following equation can be obtained:

$$E_{S,60+}[k] = 1 - \left(\frac{\frac{CC_{60+}^V|^{date\ x}}{P_{60+}^V|^{date\ x}}}{\frac{CC_{60+}^U|^{date\ x}}{P_{60+}^U|^{date\ x}}}\right) \qquad \text{(SM3-4a)}$$

or reordering,

$$\left(1 - E_{S,60+}[k]\right) = \left(\frac{\frac{CC_{60+}^V|^{date\ x}}{P_{60+}^V|^{date\ x}}}{\frac{CC_{60+}^U|^{date\ x}}{P_{60+}^U|^{date\ x}}}\right) \qquad \text{(SM3-4b)}$$

On the other hand, for the 60+ group, one can define the effectiveness against hospitalization among symptomatic individuals, considering the relative incidences

of hospitalized individuals among the symptomatic individuals grouped in the fractions vaccinated (v) or unvaccinated (u) of the study group, as follows:

$$E_{H|S,60+}[k] = 1 - \left(\frac{\frac{H_{60+}^{V}\Big|^{date\ x}}{CC_{60+}^{V}\Big|^{date\ x}}}{\frac{H_{60+}^{U}\Big|^{date\ x}}{CC_{60+}^{U}\Big|^{date\ x}}} \right) \qquad \text{(SM3-5a)}$$

or reordering,

$$1 - E_{H|S,60+}[k] = \left(\frac{\frac{H_{60+}^{V}\Big|^{date\ x}}{CC_{60+}^{V}\Big|^{date\ x}}}{\frac{H_{60+}^{U}\Big|^{date\ x}}{CC_{60+}^{U}\Big|^{date\ x}}} \right) \qquad \text{(SM3-5b)}$$

Thus, considering Equations SM3-4b and SM3-5b, the Equation SM3-3 can be modified as follows:

$$1 - E_{H,60+}[k] = \left(1 - E_{H|S,60+}[k]\right)\left(1 - E_{S,60+}[k]\right) \qquad \text{(SM3-7a)}$$

or solving for $E_{H|S,60+}[k]$,

$$E_{H|S,60+}[k] = 1 - \frac{\left(1 - E_{H,60+}[k]\right)}{\left(1 - E_{S,60+}[k]\right)} \qquad \text{(SM3-7b)}$$

Equation SM3-7b will be used for the procedures described in the manuscript.
